# S243. EFFECTS OF A VIRTUAL REALITY SOCIAL TRAINING INTERVENTION ON LONELINESS AND SOCIAL COGNITION IN PATIENTS WITH SCHIZOPHRENIA

**DOI:** 10.1093/schbul/sby018.1030

**Published:** 2018-04-01

**Authors:** Laura Hieber Adery, Megan Ichinose, Lénie Torregrossa, Heathman Nichols, Dayi Bian, Joshua Wade, Eric Granholm, Nilanjan Sarkar, Sohee Park

**Affiliations:** 1 Vanderbilt University; 2 UCSD

## Abstract

**Background:**

Deficits in social cognition and on social perception tasks are well studied and widely found in populations with schizophrenia. In addition, our work consistently replicates findings that individuals with schizophrenia report severe loneliness, significantly higher than healthy matches. Loneliness is a chronic, gnawing condition that induces distress and impedes life satisfaction and function across the spectrum of mental health. We also find social isolation impedes interpretation of social information and may lead to socio-perceptual deficits.

The present study examines the effectiveness of a novel, adaptive virtual reality simulated social exposure training intervention (see Bekele et al, 2016) in both decreasing feelings of loneliness and improving social cognitive function in individuals with schizophrenia. We investigate baseline relationships between social isolation, loneliness and social cognition abilities, as well as pre to post intervention changes in function and subjective social well-being.

**Methods:**

Fifteen medicated SZ outpatients completed 10 virtual reality social skills training sessions over the course of 5 weeks. Training sessions depicted three naturalistic social scenarios in which participants were instructed to complete 12 total social “missions” to obtain information from VR avatar characters. Prior to training and following the final training session, participants were assessed using the CogState Brief Schizophrenia Battery Social Emotional cognition task and rated loneliness using the UCLA Loneliness Scale. Independent raters conducted pre- and post-training clinical interviews to assess changes in participants’ levels of positive, negative, and overall psychiatric symptoms

**Results:**

Greater overall psychiatric symptoms were significantly correlated with higher levels of experienced loneliness, consistent with previous findings. There was a significant improvement in social emotional cognition accuracy, and a trend-level reduction in loneliness from pre-training to post-testing following social VR training.

**Discussion:**

Previous research indicates that individuals higher on the psychosis spectrum perform worse at social cognition and social perception tasks. Our own research indicates that individuals higher on the psychosis spectrum also endorse higher levels of social distress via social isolation and loneliness. The present study attempts to enhance social cognitive and interpersonal abilities of individuals with schizophrenia while decreasing loneliness by strengthening social bonds and skills using a virtual reality training game. We find that following 10 sessions of VR social training, accuracy on measures of social cognition is improved significantly, however loneliness is reduced non-significantly. These initial results demonstrate potential feasibility of a novel VR social skills training game for improving social experience for patients with schizophrenia.

